# Applications of PBPK Modeling to Estimate Drug Metabolism and Related ADME Processes in Specific Populations

**DOI:** 10.3390/pharmaceutics17091207

**Published:** 2025-09-16

**Authors:** Pavani Gonnabathula, Miao Li, Suresh K. Nagumalli, Darshan Mehta, Kiara Fairman

**Affiliations:** Division of Biochemical Toxicology, National Center for Toxicological Research, US Food and Drug Administration, Jefferson, AR 72079, USAdarshan.mehta@fda.hhs.gov (D.M.)

**Keywords:** physiologically based pharmacokinetic modeling, metabolism, disease, polymorphism

## Abstract

**Background:** Physiologically based pharmacokinetic (PBPK) models utilize computer-based simulations to predict the pharmacokinetics of drugs. By using mathematical modeling techniques consisting of differential equations to simulate blood flow, tissue compositions, and organ properties, the pharmacokinetic properties of drugs can be better understood. Specifically, PBPK models can provide predictive information about drug absorption, distribution, metabolism, and excretion (ADME). The information gained from PBPK models can be useful in both drug discovery, development, and regulatory science. PBPK models can help to address some of the ethical dilemmas that arise during the drug development process, particularly when examining patient populations where testing a new drug may have significant ethical concerns. Patient populations where significant physiological change (i.e., pregnancy, pediatrics, geriatrics, organ impairment populations, etc.) and pathophysiological influences resulting in PK changes can also benefit from PBPK modeling. Additionally, PBPK models can be utilized to predict variations in drug metabolism resulting from genetic polymorphisms, age, and disease states. **Methods:** In this mini-review, we examine the various applications of PBPK models in drug metabolism. Current research articles related to drug metabolism in genetics, life-stages, and disease states were reviewed. **Results:** Several key factors in genetics, life-stage, and disease states that affect metabolism in PBPK models are identified. In genetics, the role of CYP enzymes, genetic polymorphisms, and ethnicity may influence metabolism. Metabolism generally changes over time from neonate, pediatric, adult, geriatric, and perinatal populations. Disease states such as renal and hepatic impairment, weight and other acute and chronic diseases also can also alter metabolism. Several examples of PBPK models applying these physiological changes have been published. **Conclusions:** The utilization and recognition of these specific areas in PBPK modeling can aid in personalized dosing strategy, clinical trial optimization, and regulatory submission.

## 1. Introduction

Physiologically based pharmacokinetic (PBPK) modeling has gained more traction over the years, with a steady increase in publications and submissions to regulatory bodies [1,2]. Physiologically based pharmacokinetic (PBPK) modeling represents a mechanistic approach to understanding drug disposition through mathematical simulations of physiological processes. These models employ systems of differential equations to characterize blood flow, tissue composition, and organ-specific properties, enabling quantitative predictions of drug absorption, distribution, metabolism, and excretion (ADME) processes.

The utility of PBPK modeling extends across the drug development continuum, from early discovery through to regulatory evaluation. These models offer particular value in addressing ethical and practical challenges associated with clinical testing in vulnerable or special populations, including pregnant women, pediatric and geriatric patients, and individuals with organ impairments. In such populations, where physiological and pathophysiological changes significantly alter pharmacokinetic profiles, PBPK modeling provides a scientifically rigorous tool for estimation, which can assist with clinical studies. PBPK modeling shows additional utility over standard population PK models, particularly in special populations where clinical data is lacking or when specific organ drug concentrations are desired.

Furthermore, PBPK models serve as powerful tools for predicting interindividual variability in drug responses arising from genetic polymorphisms, age-related physiological changes, and disease-induced alterations in drug metabolism. By incorporating population-specific physiological parameters and enzyme kinetics, these models can inform dosing strategies and identify potential safety concerns across diverse patient populations.

This review examines the key applications of PBPK modeling in determining drug metabolism in specific populations, including those based on genetics, age, life stage, and disease state. Overall, understanding how these factors influence drug kinetics can aid in designing appropriate clinical trials, inform regulatory decision-making, and may even inform future personalized medicine approaches.

## 2. Drug Metabolism and Genetics in Specific Populations

### 2.1. CYP Enzyme Polymorphisms

Polymorphisms are changes in the nucleotide sequence of genes that allow for variability in specific phenotypical outcomes. Enzymes that are affected by polymorphisms can have drastic effects on metabolism, which can be subject to PBPK modeling. Polymorphisms in enzymes can cause ultrarapid, rapid, intermediate, or poor metabolism. If a particular drug is metabolized by an enzyme such as cytochrome P450 3A4 (CYP3A4) and the individual is an ultrarapid metabolizer due to polymorphisms, we could increase the rate of metabolism for that enzyme, interacting with the drug kinetically to mimic what an ultrarapid metabolizer’s activity could achieve. Additionally, a sample of the population could be examined to understand the prevalence of each type of metabolizer or single-nucleotide polymorphism (SNP) in the population for a specific enzyme, which would aid in creating more realistic population models. Several cytochromes, such as CYPs 1A2, 2D6, 2C9, and 2C19, are considered to be highly polymorphic in certain ethnicities, whereas CYP 2D6, 2A6, and 2B6 are polymorphic regardless of ethnic group [3]. UDP-glucuronosyltransferases (UGT) polymorphisms can also be modeled; however, these are less well studied. There are numerous examples of PBPK models that incorporate enzyme polymorphisms for prediction, with the overwhelming majority of the models analyzing polymorphisms in CYP2C19, CYP2D6, and CYP2C9. Additional enzymes from the CYP, UGT, carboxylesterase, N-acetyltransferase, glutathione transferase, vitamin K epoxide reductase complex, aldehyde dehydrogenase, flavin-containing monooxygenases, and thiopurine S-methyltransferase families have also been modeled using PBPK models. The frequencies of some of the enzymes and genes in various populations are shown in Table 1 [4].

### 2.2. Genetic Polymorphisms

Although genetic polymorphisms are most often thought of as affecting metabolic enzymes, there are other physiological factors to consider. The target site response or pharmacodynamic effect can differ from subject to subject. This is not explicitly related to metabolism, but it could be related to target site sensitivity or other factors. Additionally, transporters can exhibit polymorphisms, which result in genetic differences in the rate of influx or efflux [5]. This is just one of the many pathways in drug absorption, distribution, metabolism, and excretion (ADME) that can affect the pharmacokinetic profile.

### 2.3. Influence of Ethnicity

Ethnic influences in PBPK modeling can most often be attributed to genetic polymorphisms, but with variations within the confines of the appropriate distribution of the average individual in the population [6]. The most commonly considered differences are in enzyme abundances and liver volume [7]. These are genetic and physiological variations that can affect drug metabolism. The distinct metabolic profiles within various ethnic populations have been documented in multiple commercial software applications for Caucasian, Japanese, and Chinese populations [8,9]. Physiologically, variations in factors such as body composition, diet, and lifestyle can lead to additional influences on ADME. For example, social factors, such as coffee consumption or smoking-related activities, increase CYP1A2 activity, and could lead to the induction or inhibition of certain enzymes [10].

**Table 1:** Pharmacogenetic table of select enzymes or alleles showing frequencies in ethnicities as defined by ClinPGx biogeographical groups [4]. Abbreviations: cytochrome P450 enzyme (CYP), UDP-glucuronosyltransferase (UGT), solute carrier organic anion transporter (SLCO), thiopurine S-methyltransferase (TPMT), and dihydropyrimidine dehydrogenase (DPYD).

Frequencies of Enzyme and Allele Phenotypes in Biogeographical Groups										
Enzyme/Gene	Phenotype/Allele Function	African American/Afro-Caribbean	American	Central/South Asian	East Asian	European	Latino	Near Eastern	Oceanian	Sub-Saharan African
CYP2D6	Ultrarapid metabolizer	0.04	0.05	0.02	0.01	0.02	0.04	0.07	0.18	0.04
	Normal	0.54	0.67	0.58	0.53	0.49	0.60	0.57	0.64	0.46
	Intermediate	0.36	0.23	0.28	0.38	0.38	0.29	0.30	0.10	0.38
	Poor	0.02	0.02	0.02	0.01	0.07	0.03	0.02	0.00	0.02
	Indeterminate	0.04	0.02	0.10	0.07	0.04	0.04	0.04	0.09	0.09
CYP2C9	Normal	0.76	0.83	0.60	0.84	0.63	0.74	0.61	0.91	0.73
	Intermediate	0.24	0.16	0.36	0.15	0.35	0.25	0.36	0.09	0.26
	Poor	0.01	0.00	0.04	0.01	0.03	0.01	0.03	0	0.01
	Indeterminate	-	-	0.00	0.00	0.00	0.00	-	-	-
CYP2C19	Ultrarapid metabolizer	0.04	0.01	0.03	0.00	0.05	0.03	0.04	0.00	0.03
	Rapid metabolizer	0.24	0.14	0.19	0.03	0.27	0.24	0.26	0.02	0.21
	Normal metabolizer	0.33	0.63	0.30	0.38	0.40	0.52	0.45	0.04	0.37
	Intermediate or likely intermediate metabolizer	0.34	0.21	0.41	0.46	0.26	0.19	0.24	0.37	0.34
	Poor or likely poor metabolizer	0.05	0.01	0.08	0.13	0.02	0.01	0.02	0.57	0.05
	Indeterminate	0.00	-	-	0.00	-	-	-	-	-
CYP3A4	Normal metabolizer	0.21	-	0.11	0.06	0.01	0.03	0.01	-	0.23
	Intermediate or possible intermediate metabolizer	0.50	-	0.44	0.38	0.14	0.29	0.21	-	0.50
	Poor metabolizer	0.30	-	0.45	0.56	0.86	0.68	0.77	-	0.27
	Indeterminate	-	-	-	-	-	-	-	-	-
UGT1A1 allele	Decreased function	0.43	-	0.46	0.29	0.33	0.41	0.31	0.04	0.44
	Normal function	0.03	-	0.54	0.71	0.36	0.21	0.68	0.96	0.49
	Increased function	0.08	-	-	-	-	-	0.01	-	0.07
	Unknown function	0.45	-	-	-	0.31	0.38	-	-	-
SLCO1B1 allele	Increased function	-	-	-	-	0.17	-	-	-	-
	Normal function	0.99	0.76	0.93	0.87	0.66	-	0.80	1.00	0.97
	No function	0.01	0.24	0.07	0.13	0.17	-	0.20	-	0.03
	Uncertain function	-	-	0.00	0.00	0.00	-	-	-	-
TPMT allele	Normal function	0.92	-	0.98	0.98	0.95	0.94	0.97	-	0.92
	No function	0.04	-	0.02	0.02	0.04	0.05	0.03	-	0.05
	Uncertain function	0.04	-	0.00	0.00	0.00	0.00	-	-	0.02
	Unknown function	-	-	-	0.00	0.00	0.00	-	-	-
DPYD allele	Normal function	0.80	-	0.58	0.49	0.65	0.69	-	-	0.83
	Decreased function	0.02	-	0.03	0.00	0.03	0.01	-	-	0.03
	No function	0.01	-	0.01	0.00	0.01	0.00	-	-	-

## 3. Drug Metabolism in Specific Life-Stage Populations

For special populations, such as those in various life stages, PBPK modeling has several advantages over traditional clinical studies or population pharmacokinetic modeling. PBPK modeling is more mechanistic in nature. Therefore, the typical predictable physiology for a particular life stage (Table 2) or even a disease state can be incorporated via organ-specific parameterization. This allows for extrapolation from a usually data-heavy healthy adult population to a special population with less clinical data. Additionally, PBPK models can incorporate ontogeny equations, allowing for developmental changes to be incorporated into various aspects such as organ maturation, renal function, or enzyme activity. These dynamic changes can be time-dependent, which allows for greater mechanistic detail than population PK models and can reduce the patient burden. Organ-specific concentrations can be predicted with PBPK modeling, which can be important for understanding safety and efficacy in these special populations. Finally, in vitro studies, such as enzyme kinetics or transporter data, can be incorporated into PBPK modeling with in vitro to in vivo extrapolation, again making PBPK modeling a valuable tool for special populations when compared with more traditional PK routes.

### 3.1. Geriatric Populations

The geriatric population (typically those aged 65 years or older) often exhibits altered drug metabolism due to age-related physiological decline. Liver mass and hepatic blood flow progressively decrease with age, reducing the clearance of high-extraction-ratio drugs [11]. In addition, hepatic enzyme activity-particularly of CYP isoforms such as CYP1A2 and CYP2C19-can decline in older adults, while others like CYP3A4 show more variable age-related expressions [12]. Renal clearance also diminishes with aging, impacting the pharmacokinetics of renally excreted drugs.

PBPK models for elderly individuals incorporate these physiological changes to simulate altered ADME. PBPK modeling is particularly valuable in polypharmacy scenarios where older adults are at a higher risk of drug–drug interactions and adverse events [13]. These models support individualized therapeutic strategies and are increasingly used in regulatory submissions for the labeling of drugs for the geriatric population.

### 3.2. Adult Populations

Although adults (18–65 years) are considered to be the standard population in drug development, PBPK modeling is still vital in accounting for interindividual variability within this group. Factors such as genetic polymorphisms, co-morbid conditions, enzyme-inducing substances (e.g., smoking), or drug-drug interactions can significantly influence intermediate metabolic drug metabolism.

Genetic variants affecting enzymes like CYP2C19 and CYP2D6 can lead to poor, intermediate, or ultrarapid metabolism of certain drugs. PBPK modeling integrates genotypic information to predict exposure profiles, supporting personalized dosing [14].

Lifestyle factors and hepatic/renal disease states can be incorporated into adult PBPK models to simulate altered drug kinetics. These models have been applied to simulate hepatic impairment in patients with cirrhosis, thereby guiding dose recommendations for hepatically cleared drugs such as propranolol [15]. Additionally, adult PBPK models are foundational for extrapolation to special populations, such as children and pregnant women.

### 3.3. Neonatal Populations

Neonates (birth to 28 days) exhibit substantial physiological immaturity, making them particularly vulnerable to drug toxicity and therapeutic failure [16,17,18]. PBPK modeling is crucial in this age group due to the ethical and practical challenges associated with conducting clinical trials using neonates. At birth, hepatic enzyme activity is underdeveloped, with minimal expression of key enzymes, including CYP1A2, CYP2C9, and UGTs. In contrast, CYP3A7 is highly expressed in the fetal liver but is gradually replaced by CYP3A4 postnatally [19].

Renal clearance is also impaired in neonates due to immature glomerular filtration and tubular function, leading to prolonged half-lives for renally cleared drugs [20]. Moreover, a higher total body water content and lower fat stores affect drug distribution, especially for hydrophilic drugs such as aminoglycosides [21].

PBPK models have been instrumental in predicting the safe and effective neonatal dosing of antibiotics such as gentamicin and vancomycin [22]. These models incorporate enzyme ontogeny functions, dynamic changes in plasma protein binding, and neonatal-specific absorption profiles. The FDA and European Medicines Agency (EMA) increasingly rely on such models to support neonatal dosing recommendations in lieu of full-scale trials [23].

### 3.4. Pediatric Populations

Pediatric patients, spanning from infancy to adolescence, undergo rapid physiological changes that impact all aspects of drug metabolism. PBPK models must capture this developmental progression, including gastrointestinal maturation, increasing hepatic enzyme activity, and improved renal function over time.

The ontogeny of CYP enzymes plays a central role. For example, CYP3A4 activity is minimal at birth but reaches adult levels within the first year, influencing the metabolism of drugs like midazolam [24]. Similarly, the glomerular filtration rate significantly increases during infancy and stabilizes by the age of two, affecting the clearance of drugs such as amoxicillin [25].

Pediatric PBPK modeling is frequently used to extrapolate adult data for the selection of pediatric doses. The FDA encourages PBPK modeling in pediatric drug development through its Pediatric Study Plan and Written Request programs [26]. The FDA guidance and review by Johnson et. al. are exclusively dedicated to the use of PBPK modeling for pediatric dose extrapolation [27]. The review covers first-in-pediatric trials, complex formulation development, data supplementation, and difficult-to-test scenarios. One such case not covered within the aforementioned review was a mechanistic oral dapagliflozin PBPK model developed to predict accurate outcomes in monotherapy studies, drug–drug interactions with UGT inhibitors and inducers, and across both healthy and impaired populations. Additionally, an oral-clearance optimized PBPK model was created for pediatric exposure predictions and dose recommendations to match adult exposure after a single 10 mg dose [28].

### 3.5. Gestational Period (Pregnancy)

Pregnancy presents unique challenges for pharmacokinetics due to physiological adaptations that evolve throughout gestation. These include a 30–50% increase in plasma volume, a 50% rise in cardiac output, an elevated glomerular filtration rate, and significant changes in hepatic enzyme activity [29]. For example, CYP3A4 and UGT1A1 activities increase during pregnancy, whereas CYP1A2 and CYP2C19 activities decrease [30]. Such alterations can lead to underexposure or overexposure to critical medications if dosing is not adjusted. Additionally, there are safety considerations for drug transport across the placenta and fetal drug exposure.

PBPK models of pregnancy account for maternal–fetal compartments, time-dependent enzyme changes, and placental transfer rates. These models have been applied to antiretroviral therapies such as acyclovir, emtricitabine, dolutegravir, and raltegravir. PBPK models facilitate the extrapolation of drug pharmacokinetics from well-characterized populations of healthy adults to the population of pregnant women [31]. Consequently, regulatory agencies sometimes accept maternal PBPK modeling data in pregnancy labeling, especially when clinical data are lacking [32].

**Table 2:** Various ADME properties that can affect drug metabolism in several life stages, which should be considered in PBPK modeling.

Population	Neonate	Pediatric	Adolescent	Adult	Geriatric	Pregnancy
**Absorption**	Slower gastric emptying. Higher gastric pH and less acidic. Irregular peristalsis. Slow, irregular IM absorption due to decreased muscle mass and blood flow. Increased percutaneous absorption due to thinner stratum cornea and higher skin hydration.	Approaches adult values as they grow. Gastric pH decreases and becomes more acidic. Gastric emptying becomes more regular	Generally similar to adults. Gastric pH and gastric emptying are comparable to adults	Baseline for comparison. Stable gastric pH and gastric emptying. Intestinal surface area is at its maximum	Gastric emptying and peristalsis may be slower. Gastric pH may be higher (hypochlorhydria). Decreased splanchnic blood flow	Slower gastric emptying. Nausea and vomiting may affect drug absorption
**Distribution**	Higher total body water (70–80%). Lower body fat (6–13.4%). Reduced plasma protein binding due to lower albumin levels and competition from endogenous substances. More permeable blood-brain barrier.	Total body water decreases. Body fat increases. Plasma protein binding increases and approaches adult values	Body composition changes occur (e.g., rapid growth and increased muscle mass). Plasma protein binding is similar to adults	Body fat (18%) Water percentage (60%). Stable body composition. Stable Plasma protein binding	Body fat (30%); water percentage (54%). Decreased total body water. Increased body fat. Decreased lean body mass. Plasma protein binding may be reduced due to lower albumin levels	Increased total body water (up to 8 L). Increased blood volume (30–50%). Decreased plasma albumin concentration. Maternal–placental circulation can affect drug distribution. Increase in cardiac output (50%).
**Metabolism**	Immature and reduced activity for Phase I (CYP450) and Phase II (conjugation) enzyme systems	CYP450 enzymes may be more active than in adults, leading to faster drug clearance for some drugs	Metabolic activity generally similar to adults but some enzyme systems may be at peak activity	Stable hepatic metabolism, which serves as the reference point for drug clearance	Phase I (CYP450) metabolism is generally decreased, such as CYP1A2 and CYP2C19. Phase II (conjugation) metabolism is less affected. Decreased hepatic blood flow.	CYP450 activity variable (induced, inhibited, or no change). Increase in CYP3A4 and UGT1A1. Decrease in CYP1A2 and CYP2C19Increased hepatic blood flow.
**Excretion**	Low and immature glomerular filtration rate (GFR) and tubular secretion	GFR and tubular secretion mature, often becoming more efficient than in adults.	Renal function similar to adults	Stable renal function, which serves as the reference point for drug clearance.	GFR and renal blood flow progressively decline. Decreased tubular secretion	Significantly increased GFR and renal blood flow, resulting in faster elimination for some drugs.
**References**	[16,17,18,19,20,21,22]	[16,19,21,22]		[19]	[11,12,19]	[26,27]

## 4. Disease States

The most common disease states in the human population are renal impairment, hepatic impairment, heart failure, and obesity. The frequency of published journal articles about PBPK disease-state models correlates to the extent of these diseases, which are discussed in more detail below.

### 4.1. Renal Impairment

When considering renal impairment, one must consider how this affects the pharmacokinetics of the drug when building the pharmacokinetic model. If the drug or its metabolites are primarily renally cleared, there will be a direct effect on the plasma or target site concentration. Additionally, although this review mainly focuses on renal-specific pathways, it is also worthwhile to consider the potential changes that renal disease may have on non-renal clearance mechanisms [33,34]. Renal impairment can also alter drug distribution via changes in fluid balance and protein binding [35,36]. The accumulation of toxins may also further affect transporters and metabolism. There are numerous reviews and disease- or drug-specific examples of PBPK modeling and renal impairment [13,37,38]. PBPK models account for modifications in renal function by incorporating a reduced glomerular filtration rate and altered transporter expression, such as organic anion transporters and organic cation transporters [39]. Drugs primarily eliminated via renal pathways (e.g., gabapentin) have been modeled to guide dosage in patients with varying stages of chronic kidney disease [40]. The integration of both renal and hepatic impairment in combined disease models is also gaining attention, especially in geriatric or polypharmacy patients [41].

### 4.2. Hepatic Impairment

The liver is the main site for metabolism. Thus, for the many drugs metabolized by the liver, hepatic impairment can cause a reduction in metabolism (e.g., CYP [42] and UGT [43] enzymes) and alterations in drug transport as the expression and function of transporters within the liver can also be impaired by a cirrhotic liver. PBPK models utilize Child-Pugh scores to adjust liver enzyme levels and blood flow, thereby aiding in the prediction of changes in first-pass metabolism and clearance [44]. For example, PBPK simulations of propranolol in patients with cirrhosis closely match clinical observations, demonstrating the model’s reliability [15].

The liver is also responsible for producing many plasma proteins [45,46,47], like albumin, which bind to most drugs. An alteration in albumin levels could affect protein binding, effectively altering drug distribution, volume, and clearance in the plasma, which directly affects metabolism. Finally, hepatic impairment can result in reduced liver blood flow, which could affect clearance and drug delivery, especially for high-extraction drugs. Compared with renal impairment, fewer PBPK publications exist, specifically for the exploration of hepatic impairment or cirrhosis [43,44,48,49,50]. Individual drugs comprise most of the publication domain. Amongst the disease states for liver impairment, searches for NASH and NAFLD increase the discoverable yield of publications on this subject; however, they are still few compared with those for renal impairment.

### 4.3. Body Mass Index and Weight

Body mass index (BMI) and weight are two ways to assess patients or research subjects for dosing purposes. BMI is often used to determine if a person is underweight, of normal weight, overweight, or obese.

There are even fewer searchable publications on obesity PBPK models compared with hepatic or renal impairment. However, PBPK models could be helpful in this area, depending on the type of drug being researched. Obesity can lead to an increase in adipose tissue, altered organ sizes and blood flow, and changes in renal function as well as alterations in enzyme activity, which may affect half-life, hepatic and renal metabolism, clearance, and volume of distribution [51]. Therefore, using PBPK models, especially for drugs that have a high volume of distribution or are more lipophilic, would likely have greater effects in obese individuals than in normal-weight subjects, for which a PBPK model could be used to predict ADME more accurately. PBPK models utilize allometric scaling principles to account for weight-related changes in pharmacokinetics [52]. In obese patients, alterations in the volume of distribution and clearance are frequently observed, necessitating dose adjustments [53]. For lipophilic drugs like midazolam and propofol, PBPK models show significantly altered kinetics in obese patients, necessitating weight-based or adjusted body weight dosing [54,55].

Predictions about drug disposition could better inform dose adjustments in this population for dose optimization and potentially in clinical trials. However, search results in this area indicate that data are lacking [51], which is unfortunate considering the high level of obesity and overweightness in the United States and other nations worldwide.

### 4.4. Other Diseases (Chronic and Acute)

Due to the complexity and infinite number of potential disease states, we briefly cover some of the more frequently published PBPK models and considerations for some of those disease states. As many physiologies can exist that may influence PK outcomes, co-morbidity PBPK models must be considered to properly capture various metabolic processes.

#### 4.4.1. Chronic Diseases

Chronic disease modeling in PBPK modeling involves incorporating disease-specific physiological and biochemical alterations that affect drug metabolism. Chronic conditions, such as diabetes, cancer, cardiovascular disease, and autoimmune conditions, often result in long-term physiological changes that can affect drug metabolism by significantly modifying metabolic enzyme activity, organ function, blood flow distribution, and plasma protein levels, all of which influence drug ADME. PBPK modeling provides a framework to assess these changes. This approach supports precision medicine by tailoring drug therapy to individual patient conditions.

Diabetes mellitus is associated with altered gastric emptying, variations in plasma protein binding, and changes in the expression of CYP enzymes, particularly CYP2E1 [56]. PBPK models are used to simulate these parameters and predict the kinetics of antidiabetic drugs, such as metformin, as well as their interactions with co-medications [57].

Tumors and cancer treatments can influence hepatic metabolism, gastrointestinal function, and systemic inflammation. Additionally, the combination of cancer with various disease states or life stages, such as pregnancy [58], may make it difficult to gather information during clinical trials. PBPK modeling is used in oncology to optimize chemotherapy dosing and characterize interpatient variability in drug responses due to tumor-induced cachexia or liver metastases [59].

Heart failure can alter organ blood flow and cardiac output, leading to issues with organ perfusion. Although few PBPK models exist in this area, PBPK models have predicted altered kinetics for this population. For example, captopril dose estimations in patients with chronic heart failure and chronic kidney disease were successfully predicted using PBPK modeling [60].

Chronic inflammatory conditions, such as those seen in rheumatoid arthritis or inflammatory bowel disease, lead to a downregulation of hepatic CYP enzymes through the action of pro-inflammatory cytokines. This modulation has a significant impact on drug metabolism [61]. PBPK models integrate cytokine profiles to simulate PK changes and inform immunosuppressant dosing [62].

#### 4.4.2. Acute Diseases

Acute disease modeling in PBPK modeling involves incorporating transient but significant physiological and biochemical changes that affect drug metabolism. Acute conditions, such as sepsis, inflammation, or acute liver and kidney injuries, can rapidly alter enzyme activity, organ function, and blood flow, leading to dynamic changes in ADME. These short-term changes pose challenges for PBPK modeling, but these can be integrated.

Infectious diseases and sepsis lead to systemic inflammation, which results in decreased hepatic and renal functions. This affects drug transporters, such as P-glycoprotein and breast cancer resistance protein, and reduces CYP activity [63]. For example, during acute inflammation or infection, the release of cytokines can suppress CYP activity, thereby reducing drug metabolism and increasing systemic drug exposure, which may lead to toxicity [61]. PBPK models are utilized to simulate the kinetics of antibiotics and antimicrobials in critically ill patients who may have altered volumes of distribution and impaired clearance [64,65].

Acute liver failure is characterized by abrupt hepatic dysfunction, which significantly reduces phase I and II metabolic capacity. As discussed above, PBPK models can simulate reduced enzyme activity (e.g., loss of CYP3A4) and altered hepatic blood flow to predict the toxicity risk from standard dosing regimens [42]. Using patient-specific clinical data, such as alanine transaminase, bilirubin, and creatinine clearance, for real-time model adaptation may improve the applications of PBPK modeling in acute care settings. Similarly, acute kidney injury can impair renal clearance, leading to drug accumulation. PBPK models integrate these acute disease-specific physiological changes to predict their impact on drug disposition, allowing for real-time adjustments in dosing and therapy to ensure drug safety and efficacy in patients experiencing sudden physiological disturbances [13,66].

## 5. Discussion

### 5.1. Personalized Dosing Strategy

A personalized dosing strategy in PBPK modeling tailors drug administration to an individual’s specific physiological and metabolic characteristics, thereby improving therapeutic efficacy while minimizing adverse effects. PBPK models can integrate patient-specific factors such as genetic polymorphisms into drug-metabolizing enzymes (e.g., CYP variants), age (e.g., pediatric, adult, or geriatric), organ function (e.g., liver or kidney impairment), body composition (e.g., BMI or fat-to-lean mass ratio), sex (e.g., male or female), and disease states (e.g., chronic or acute conditions) [67]. These models predict how variations in metabolism affect drug ADME, enabling precise dose adjustments for different subpopulations that may not have been studied in clinical trials during drug development, including pediatric and geriatric populations and individuals with metabolic disorders. Although there is still work to be undertaken, simulating real-world drug behavior in diverse patient groups could enable PBPK-based personalized dosing strategies to enhance drug safety, optimize therapeutic outcomes, and support precision medicine approaches in clinical practice [67].

### 5.2. Clinical Trial Optimization

As previously discussed, PBPK modeling allows the generation of virtual subpopulations that can be utilized in clinical trial optimization to design more efficient and targeted studies, thus reducing trial costs and increasing drug development success rates. The integration of key physiological factors, such as genetic polymorphisms, enzyme activity (e.g., CYP variations), organ function (e.g., liver or kidney impairment), and disease states, enables PBPK models to simulate how drugs are metabolized across diverse populations. These simulations help to identify potential metabolic differences before clinical trials begin, allowing for optimized dosing strategies, refined inclusion criteria, and a reduced risk of adverse effects, and minimizing the need for extensive trials in these groups [67]. By predicting drug interactions, metabolism variability, and optimal dose adjustments, PBPK modeling can enhance trial designs, ensuring safer, more efficient, and more personalized drug evaluations.

### 5.3. Regulatory Submission

Regulatory submissions that utilize PBPK modeling to predict subpopulation pharmacokinetics play an important role in demonstrating how metabolism influences drug disposition, safety, and efficacy. Although most new drug application submissions to the FDA still primarily focus on drug-drug interactions, susceptible subpopulation models for life stages (e.g., children), organ impairment, and genetic polymorphisms make up a smaller percentage of submissions [1,2]. By predicting metabolic variability across diverse patient groups, PBPK models help to refine clinical trial data, reducing the need for certain in vivo studies and providing supporting evidence for the safe and effective use of drugs in populations with altered metabolism. This approach enhances regulatory decision-making by ensuring that metabolism-related risks are thoroughly assessed before market approval.

## 6. Conclusions

PBPK modeling to estimate drug metabolism in specific populations, particularly in subpopulations, is a definitive step for improving patient, research, and clinical trial outcomes. Several areas of risk can be identified and mitigated, which can enhance subjects’ and patients’ experience, safety, and drug efficacy. Additionally, the construction and exploration of subpopulations using PBPK models enable a more granular examination of the knowledge gaps that may exist within those populations.

## 7. Future Directions

**Figure 1:**
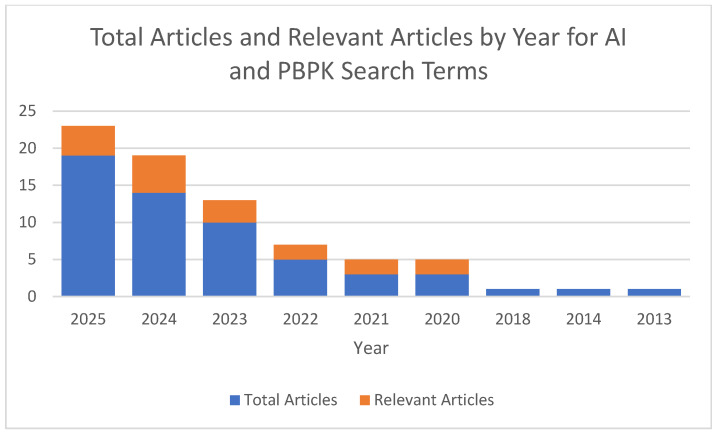
Graph of total articles and relevant articles generated from PubMed using the search query (“artificial intelligence”) AND (PBPK OR “physiologically based pharmacokinetic”).

PBPK modeling is already recognized as a new alternative method that is gaining traction and credibility depending upon its application. Several groups are adding layers to the usefulness and testing the intersection of artificial intelligence and PBPK modeling. A recent search for (“artificial intelligence”) AND (PBPK OR “physiologically based pharmacokinetic”) indicated that approximately 55 articles were indexed on PubMed from 2013 to August 2025 [68]. A further analysis of these articles revealed that only 7 utilized artificial intelligence with PBPK modeling and 10 reviewed the utility of AI and PBPK modeling together. Since the first publication mentioning artificial intelligence and PBPK modeling, there has been a steady increase in publications incorporating both areas (Figure 1), from general discussions on the integration of AI and machine learning into PBPK models [69] to AI-assisted PBPK models [70]. With the advent of more powerful computers, machine learning models, and access, there is undoubtedly an ocean of opportunity waiting to be explored in these two areas.

## Abbreviations

The following abbreviations are used in this manuscript:

PBPK	Physiologically Based Pharmacokinetic
ADME	Absorption Distribution Metabolism Excretion
FDA	Food and Drug Administration
EMA	European Medicines Agency
NASH	Non-Alcoholic Steatohepatitis
NAFLD	Non-Alcoholic Fatty Liver Disease
